# Supplementary material for: Effect of Application Amounts on In Vitro Dermal Absorption Test Using Caffeine and Testosterone
Source: Pharmaceutics. 2021 Apr 30;13(5):641. doi: 10.3390/pharmaceutics13050641 (PMC8147129; doi:10.3390/pharmaceutics13050641)
Supplement: Supplementary file 1 [file pharmaceutics-13-00641-s001.zip › pharmaceutics-1175502-SI.pdf]

pharmaceutics

Supplementary Materials: Effect of Application Amounts on In Vitro Dermal Absorption Test Using Caffeine and Testosterone

Jueng-Eun Im, Hyang Yeon Kim, Jung Dae Lee, Jin-Ju Park, Kyung-Soo Kang and Kyu-Bong Kim

Table S1. Intra-day and inter-day accuracy and precision of caffeine.

Matrix	Concentration (μg/mL)	Intra-Day		Inter-Day	
		Accuracy [1] (%)	Precision [2] (%)	Accuracy [1] (%)	Precision [2] (%)
WASH [3] (cream)	0.05	90.8	14.7	96.7	18.7
	0.15	93.9	8.8	97.8	8.4
	0.60	101.8	2.0	102.5	2.3
	1.20	102.9	2.0	103.7	2.5
WASH [3] (solution)	0.05	104.8	7.3	94.8	14.8
	0.15	96.3	3.7	95.9	3.8
	0.60	105.5	5.1	103.1	4.3
	1.20	105.6	3.4	102.8	3.8
S.C [4]	0.05	104.0	7.7	98.4	9.9
	0.15	98.3	6.6	98.0	11.4
	0.60	103.9	1.9	108.5	5.9
	1.20	107.6	3.2	102.7	5.6
SKIN [5] (rat)	0.05	100.8	11.6	94.0	18.0
	0.15	89.7	5.4	91.5	5.7
	0.60	111.7	3.2	100.5	9.4
	1.20	102.4	2.8	102.8	7.7
SKIN [5] (Micropig®)	0.05	98.0	7.2	102.3	15.0
	0.15	97.9	4.0	95.7	7.2
	0.60	99.1	6.8	100.3	4.5
	1.20	99.1	5.9	102.4	5.4
R.F [6]	0.05	105.2	12.1	95.3	14.6
	0.15	98.1	8.2	94.7	5.8
	0.60	102.8	1.4	100.9	3.8
	1.20	100.2	4.5	100.5	4.0

[1]Accuracy: (mean concentration analyzed / nominal concentration) × 100 (%), [2]Precision: relative standard deviation, [3]WASH: remaining formulation was rinsed using alcohol swabs, [4]S.C: stratum corneum, [5]SKIN: dermis and epidermis, [6]R.F: receptor fluid.

Table S2. Intra-day and inter-day accuracy and precision of testosterone.

Matrix	Concentration (μg/mL)	Intra-Day		Inter-Day	
		Accuracy [1] (%)	Precision [2] (%)	Accuracy [1] (%)	Precision [2] (%)
WASH [3] (cream)	0.3	105.5	7.6	110.4	9.1
	0.9	93.6	12.8	95.1	8.5
	3	110.9	6.6	110.8	5.1
	8	102.4	6.3	105.8	6.5
WASH [3] (solution)	0.3	111.1	4.0	107.7	7.7
	0.9	97.3	12.0	93.5	7.5
	3	103.9	6.5	105.6	4.2
	8	98.6	5.5	102.2	4.4
S.C [4]	0.1	112.6	14.1	108.3	9.9
	0.3	106.9	2.9	103.6	5.5
	3	102.7	3.0	107.3	5.2
	8	103.4	3.2	98.6	8.1
SKIN [5] (rat)	0.1	100.4	7.8	105.1	9.0
	0.3	107.8	9.9	94.9	12.3
	3	110.0	1.7	107.9	2.5
	8	106.5	3.3	105.8	5.1
SKIN [5] (Micropig®)	0.1	96.3	10.1	104.4	11.7
	0.3	102.9	6.9	106.9	6.1
	3	108.0	3.4	107.8	5.7
	8	105.5	6.6	108.2	4.2
R.F [6]	0.1	92.4	10.4	107.3	14.7
	0.3	106.6	8.0	108.7	5.6
	3	114.4	3.7	110.6	5.9
	8	88.4	1.8	102.2	11.1

[1]Accuracy: (mean concentration analyzed / nominal concentration) x 100 (%), [2]Precision: relative standard deviation, [3]WASH: remaining formulation was rinsed using alcohol swabs, [4]S.C: stratum corneum, [5]SKIN: dermis and epidermis, [6]R.F: receptor fluid.
